# Paecilomyces pyelonephritis in a patient with urolithiasis

**DOI:** 10.4103/0970-1591.32075

**Published:** 2007

**Authors:** K. Sriram, Mary S. Mathews, Ganesh Gopalakrishnan

**Affiliations:** Department of Urology, Christian Medical College, Vellore, Tamilnadu, India; * Department of Microbiology, Christian Medical College, Vellore, Tamilnadu, India

**Keywords:** Nosocomial urinary tract infections, *Paecilomyces variotii*, pyelonephritis

## Abstract

Fungal organisms are increasingly implicated in nosocomial urinary tract infections. Although *Candida, Mucor* and *Aspergillus* are the most commonly identified species, rare fungi are also occasionally observed to infect humans. Misidentification of the organism could result in treatment with an inappropriate antifungal agent, which could result in a florid fungal pyelonephritis. We report the occurrence of fungal pyelonephritis in a patient with stone disease secondary to *Paecilomyces variotii*. This case report emphasizes the need for an accurate identification of the organism and early and appropriate treatment.

## INTRODUCTION

Fungal organisms are a major source of nosocomial urinary tract infections in the present era of endo-urological procedures. We report a case of *Paecilomyces* pyelonephritis in a patient with urolithiasis, who had undergone multiple endoscopic procedures and had antifungal treatment in the past. Once an appropriate diagnosis of *Paecilomyces* infection was made and he was started on proper antifungal agents, he became afebrile and subsequent urine cultures were sterile. This report emphasizes the need for appropriate identification and treatment of the organism and also for adopting strict asepsis during all routine endo-urological procedures.

## CASE REPORT

A 49-year-old male, diabetic for the past 15 years, on oral hypoglycemic agents, was evaluated in September 2005 elsewhere for recurrent episodes of bilateral loin pain associated with high-grade fever, chills and rigors. Intravenous urogram revealed bilateral renal calculi and right lower ureteric calculus. He underwent right ureteroscopy (URS) and intracorporeal lithotripsy with bilateral double J (DJ) stenting in September 2005 and subsequently had two sittings of shock wave lithotripsy (SWL) for left renal calculus. He continued to have febrile episodes following DJ stents removal. He then had bilateral retrograde intrarenal surgery (RIRS) and DJ stenting on the left side in December 2005 as he had a few residual stones in the left kidney. As urine culture was positive for a *Candida*-like organism, he was also then treated with oral fluconazole, 200 mg once a day for three weeks. The febrile UTI however persisted and hence he was referred to us for further management.

On admission, he was febrile, tachycardic and normotensive. Left renal angle was tender. The packed cell volume was 35% with neutrophilia but no leukocytosis. Serum creatinine was marginally elevated (1.6g %). Urine analysis showed numerous leukocytes and 8-10 erythrocytes/ HPF. Serum sodium was 131 mmol/L and serum potassium: 4.2 mmol/L. HbA1C was 7.3%. Suprapubic urine culture yielded fungal growth, which was identified as *Paecilomyces variotii*. Ultrasound of the kidneys showed bilateral small renal calculi with focal cortical hypodense areas in both the kidneys. The CT scan abdomen showed a 27 × 27 mm simple cyst in the interpolar region of the right kidney, 6 mm calculi in the lower calyx and interpolar region of the right kidney. The left kidney showed evidence of lobar nephronia with perinephric stranding and a 15 × 16 mm cortical cyst in the upper pole with a 6 mm calculus in the renal pelvis. The left side DJ stent was removed and sent for fungal culture, which also yielded a growth of *Paecilomyces variotii*. He was started on intravenous Amphotericin B, which was given at a dosage of 1 mg per kg per day for four weeks, following which he became afebrile. A complete eradication of *Paecilomyceal* infection was confirmed by two repeat urine C/S samples done one week after the completion of the antifungal course, which were sterile.

## DISCUSSION

**Figure 1:**
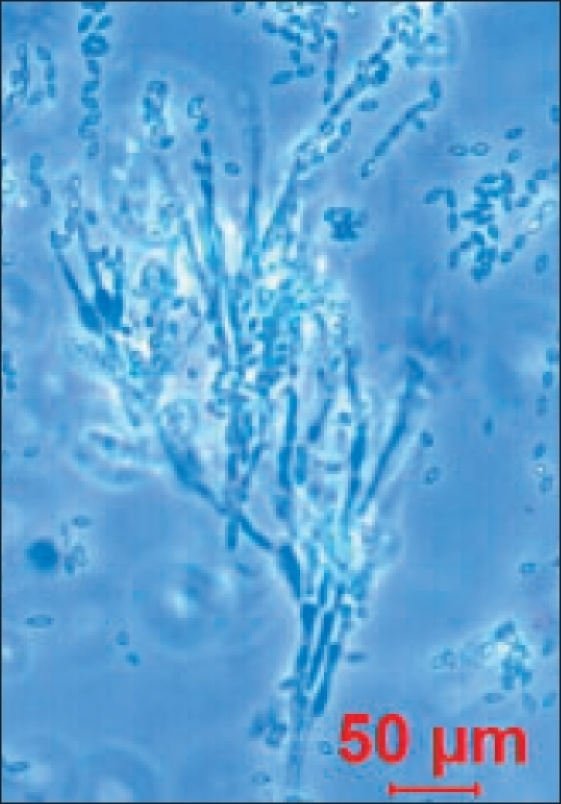
Microphotograph showing *Paecilomyces variotii* with septate hyphae in a penicillius arrangement, the phialides having a wide base, tapering to a long, slender neck (Lacto-Phenol Cotton Blue stain ×40 magnification)

**Figure 2:**
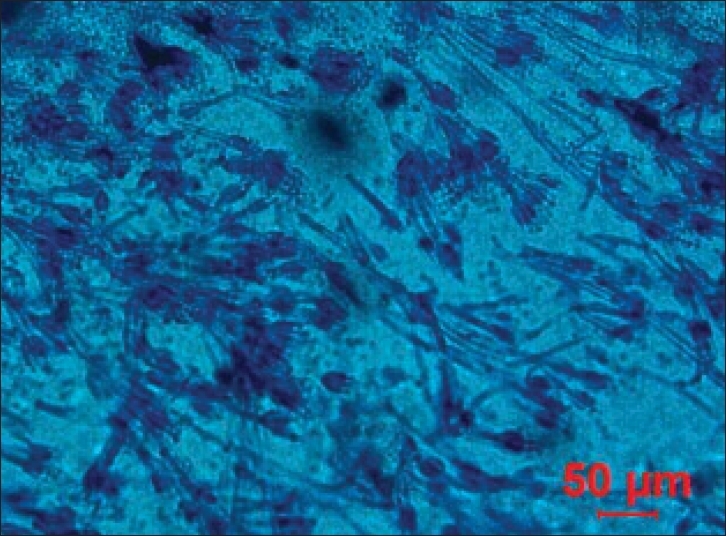
Microphotograph showing the more common *Penicillium* species with slender septate hyphae (Lacto-Phenol Cotton Blue stain ×40 magnification)

*Paecilomyces* species are saprophytic fungi that are usually recovered from soil and decaying vegetation. *P. variotii* and *P. lilacinus* are the two ubiquitous species of the genus and also the most frequently involved in human infections.[1] The colonies of *Paecilomyces variotii* are initially flat and floccose. The texture changes to powdery and the color of the isolate is yellow-brown. Microscopically, the hyphae are hyaline and septate. Long chains of elliptical conidia are produced from phialides that have long narrow necks [Figure 1]. The phialides developing from metulae at the tips of the conidiophores - the “Penicillius” arrangement may cause this fungus to be confused with the more common *Penicillium* [Figure 2]. *Paecilomyces* differs from *Penicillium* in several aspects (1) penicilli are less well defined; (2) conidia are rarely of the symmetrical, spherical to ellipsoidal shape characteristic of *Penicillium*; (3) conidial mass is bright colored and rarely green or never blue; (4) phialides are longer than those of *Penicillium*.[2] Our isolate was *Paecilomyces variotii,* identified by its characteristic colonial morphology showing chains of single-celled phialoconidia (ameroconidia) produced in basipetal succession from a phialide and the characteristic yellow-brown color of the pigment. As microscopic features resemble the *Penicillium* species very closely, inexperienced laboratory workers who are not conversant with this uncommon fungus can easily misidentify it.

*Paecilomyces variotii* has been reported to infect men, especially those who are diabetic or immunocompromised or in the presence of a foreign body. The first case of *Paecilomyces* of the genitourinary tract was reported by Sherwood *et al* in 1983 in which a resolution of infection occurred with correction of obstructive uropathy alone.[3]

The most common *Paecilomyceal* species that were known to infect man,*P. variotii* and *P. lilacinus*, show very clear differences in the *in vitro* susceptibility to the currently used antifungal agents. Aguilar *et al* reported that *P. variotii* was most susceptible to Amphotericin B while showing poor susceptibility to fluconazole.[4] If proper species identification is not done, these patients will be treated with inappropriate antifungal agents. This could result in a persistent fungal pyelonephritis, in the setting of an already existing immunocompromised status, as in our patient.

The standard treatment for *Paecilomyces* species is either amphotericin B alone or in combination with flucytosine or azoles. The failure rate with amphotericin B is as high as 40%, which only indicates that the appropriate treatment regimen for these organisms has not been arrived at.[1] *In vitro* studies by Ortoneda *et al* have shown that the combination of terbinafine with azoles has the highest percentage of synergistic effect against *Paecilomyces* species. Prevention and control of *Paecilomyceal* fungal infection requires a high index of clinical suspicion, application of most sensitive diagnostic modalities and specifically directed use of antifungal agents. Moreover, it is very important to maintain strict asepsis during endoscopic procedures. In endo-urological procedures, there is a very high chance of a breach of sterile technique and this could result in the development of the nosocomial infection. Careful attention towards each of these areas will go a long way towards early and appropriate treatment, which in turn improves patient management and successful eradication of such nosocomial infections.

## References

[1] Ortoneda M, Capilla J, Pastor FJ, Pujol I, Yustes C, Serena C (2004). *In vitro* interactions of approved and novel drugs against *Paecilomyces* spp. Antimicrob Agents Chemother.

[2] Thai Entomogenous *Paecilomyces*: A comparison with CBS *Paecilomyces* strains: National Center for Genetic Engineering and Biotechnology, National Science and Technology Development Agency, 73/1 Rama VI Rd, Rajdhevee, 10400 Bangkok, Thailand. [Last accessed on 2006 Nov 10].

[3] Sherwood JA, Dansky AS (1983). *Paecilomyces* pyelonephritis complicating nephrolithiasis and review of Paecilomyces infections. J Urol.

[4] Aguilar C, Pujol I, Sala J, Guarro J (1998). Antifungal susceptibilities of Paecilomyces species. Antimicrob Agents Chemother.

